# Tissue factor potentiates adherence of breast cancer cells to human umbilical vein endothelial cells under static and flow conditions

**DOI:** 10.1080/19336918.2021.1898709

**Published:** 2021-03-18

**Authors:** Yanling Jin, Wei Liu, Fengxia Wang, Min Wang, Kai Xu, Aijun Yang, Chenyu Wang, Lihan Zhang, Fangfang Zhang, Min Li

**Affiliations:** a Institute of Pathology, School of Basic Medical Sciences, Lanzhou University, Lanzhou, China; b First Affiliated Clinical Hospital, Lanzhou University, Lanzhou, Gansu, China; c Department of Integrated Traditional Chinese and Western Medicine, The Affiliated Cancer Hospital of Zhengzhou University, Zhengzhou, Henan, China; d Gansu Provincial Key Laboratory of Preclinical Study for New Drug Development, Lanzhou University, Lanzhou, China

**Keywords:** Tissue factor, breast cancer, endothelium, adhesion, β_1_ integrin

## Abstract

Tissue factor (TF) has been extensively studied for tumor metastasis, but its role in mediating cancer cell adhesion to vasculature remains unknown. This study aimed to measure the ability of TF to mediate the adhesion of breast cancer cells to human umbilical vein endothelial cells (HUVECs). MDA-MB-231 cells expressed the highest TF level and adhered more to HUVECs under static and flow conditions, a neutralizing TF antibody abolished the enhanced adhesion of MDA-MB-231 cells to HUVECs. Recombinant human soluble TF (rTF) bonded β1integrin on HUVECs surfaces, β1 or α3integrin antibody combined with TF antibody abolished more cell-cell adhesion. These data suggested that TF mediated adhesion of breast cancer cells to endothelial cells may rely on β1integrin on HUVECs surfaces.

## Introduction

The recurrence and metastasis are the main causes of death for patients with breast cancer [1], in part because recurrent and metastatic breast cancer cells respond poorly to chemotherapy [2]. This is particularly true for patients with triple-negative breast cancer (TNBC), who also carry a high risk for relapse within 3 years after surgery and a low survival rate after metastatic relapse [3]. TNBC is defined as breast cancer cells that lack the expression of estrogen receptor (ER), progesterone receptor (PR), and epidermal growth factor receptor 2 (HER2) [4]. The blockage of tumor cells interaction with endothelial cells (ECs) could therefore reduce the metastasis of breast cancer cells to improve the outcome of patients [5]. There have been extensive efforts to identify critical molecules and pathways that mediate the metastasis of breast cancer cells through ECs [6–8] .

Cancer metastasis involves sequentially cell proliferation, the local invasion of detached cells, transendothelial intravasation and extravasation of cancer cells in a target organ, resulting in the formation of secondary neoplasm [9,10] . The adhesion of cancer cells to endothelial cells is therefore a key step in the metastatic process and is believed to be initiated by E- and P-selectin expressed on activated endothelial cells interesting with the tetrasaccharide carbohydrates attached to O-glycans on the surface of cancer cells [11].The adhesion between Acute Myeloid Leukemiacells and endothelial cells requires functional P-selectin and E-selectin [12].This initial contact between ECs and cancer cells is further enhanced by integrins on cancer cells, interacting with intercellular adhesion molecule 1 on ECs [13,14].

These ligand-receptor interactions trigger cancer cell extravasation through the endothelial cell-cell junction. Recent studies have further identified tissue factor (TF) as a new mediator for the cancer metastasis [15,16] .

TF is a 47KD transmembrane glycoprotein that is constitutively expressed on extravascular cells, such as muscle cells, brain cells, and kidney cells [17]. TF is also expressed on lymphocytes and monocytes, but only after induction by traumatic or inflammatory stimulations [18–20] . TF primarily functions to initiate the extrinsic coagulation at sites of vascular injury, but has been increasingly identified as a risk factor for cancer metastasis and prognosis [21–23], because of its ability to promote cell proliferation, migration, invasion, metastasis and angiogenesis. TF does so by increasing the expression of the anti-apoptotic genes such as Bcl-2 and angiogenesis factors such as VEGF and IL-8 [24–26], by controlling the state of tumor dormancy, and by promoting the genomic alteration of cancer cells [27] . A TF splice variant, which lacks the cytoplasmic domain, has been shown to mediate the adhesion of monocytes to microvascular endothelial cells (MVECs) by ligating the β_1_ integrin on MVECs [28], which is known to mediate the interaction between cancer cells and endothelial cells [29] . Furthermore, the cytoplasmic domain of full length TF also interacts with the cytoskeletal actin-binding proteins to mediate the adhesion and migration of human bladder carcinoma cells [30], likely by regulating the cytoskeletal movement of cancer cells. Despite of these early studies, the question remains as whether tumor-derived TF mediates the adhesion of tumor cells to endothelial cellsby ligating integrin. Here, we reported that TF expressed on MDA-MB-231 breast cancer cells interacted with endothelial integrin to strengthen the tumor/endothelial adhesion.

## Materials and methods

### Cell culture

Cells from human breast cancer MDA-MB-231, HS-578 T, MCF-7 lines and human umbilical vein endothelial cell (HUVECs) were purchased from China Center for Type Culture Collection (CCTC, Beijing). Among the three breast cancer lines, MDA-MB-231 is a well-established and characterized TNBC cell line that is widely used to study the molecular mechanism of breast cancer metastasis [31]. These cells were cultured in Dulbecco’s modified Eagle’s medium (DMEM, Hyclone) containing 10% fetal bovine serum (FBS, Biological Industries). HUVECs were maintained in Dulbecco’s modified Eagle’s medium/Hamm’s nutrient mixture F12 (DMEM/F12; Hyclone) supplemented with 10% FBS. All cells were cultured at 37°C with 5% CO2 until 70%- 80% of confluence before experiments, which were performed using cells within 15 passages.

### Immunohistochemistry

Cells were scraped off flasks after rinsing two times by PBS, centrifuged 1,000 rpm to obtain cell pellet, and fixed in 4% paraformaldehyde (Solarbio,P1110) at 4°C overnight. Cell pellets were collected and embeded in filter paper, and further processed for paraffin embedding. Subsequent staining is performed according to immunohistochemical staining steps, sections were incubated with rabbit anti-human TF polyclonal antibody (1:100 dilution, Abcam, Ab104513) and rabbit anti-human β_1_integrin polyclonal antibody (1:300 dilution, GeneTex, GTX128839) and incubated at 4°C overnight, after washing, and then incubating with an HRP- conjugated IgG for 15 min, a chromogenic substrate DAB solution was incubated for 5 min, then slides were counterstained with hematoxylin.

### Immunoblots

Cells were harvested, precipitated, and lysed in a cell RIPA Lysis Buffer (Sigma-Aldrich) supplemented with protease inhibitors (Sigma-Aldrich). The cell lysates were centrifuged to remove cell debris and their protein concentration was determined by the Bradford assay. The Cell lysates (50 μg total proteins/lane) were separated by 10% SDS-polyacrylamide gel electrophoresis and electro-transferred to polyvinylidene fluoride (PVDF) membranes. The membrane was blocked with 5% nonfat dry milk in Tris-buffered saline-Tween 20 (TBST, pH 7.6) for 1 hrs at room temperature, incubated with rabbit anti-human TF polyclonal antibody (Abcam, Ab104513), mouse anti-human -β-Tublin monoclonal antibody (Immuno Way, M3030) overnight at 4°C. The membrane was washed and incubated for 1 h with appropriate fluorescently-conjugated secondary antibodies (1:10,000 dilution, Licor, 926–32,211). The antibody binding was detected using the Odyssey detection system (Licor Biosciences, Nebraska, US).

### Flow cytometric analysis of protein expression on the cell surface

Cells were collected, fixed in 4% paraformaldehyde for 10 min, and washed twice with PBS, after blocked with 2% BSA for 20 min, cells were incubated with primary antibody (TF(Abcam, Ab104513),fibronectin (ProteinTech, 15,613-1-AP), α_3_ integrin (ProteinTech,66,070-1-Ig), and β_1_ integrin antibody(GeneTex, GTX128839),1:100 dilution) on ice at 4°C for 30min, then cells were washed with PBS two times, and incubated with FITC-Goat anti-mouse secondary antibody (1:1000 dilution, immuno way,RS0003) or FITC-Goat anti-rabbit secondary antibody(1:1000 dilution, immuno way,RS0004) at 4°C for 30 min and protected from light. After washed twice with PBS, cells were examined by using flow cytometry (BD Biosciences, USA).

### Cell adhesion assay

The adhesion of breast cancer cells to HUVECs was measured under static and flow conditions. For the static assay, cells (4 × 10^5^cells/mL) were stained with Hoechst 33,342 (10 μg/mL, Solarbio, B8040) for 5 min at room temperature in the dark and then washed two times with culture media. The cells were first evaluated for viability using trypan blue staining to ensure that > 90% of cells were viable. They were then incubated with confluent HUVECs for 30 min at 37°C and 5%CO2. Non-adherent cells were removed by washing with PBS, four random fields of cells were recorded under a phase-contrast inverted fluorescence microscope (NiKon Diaphot 330, Japan) excluding the edges. To measure cell adhesion to HUVECs under flow condition to mimic the blood flow, we used a parallel-plate flow chamber system (Glycotech, Rockville, MD), as described previously [12]. Briefly, the flow chamber was washed with serum-free medium andproceeded a monolayer of HUVECs for 24 hrs at 37°C. Breast cancer cells (5 × 10^5^–1 × 10^6^ cells/mL)suspended in 1 mL cell culture medium with 0.05%BSA and prestained with Hoechst 33,342 (10 μg/mL), and perfused 30 minthrough the chamber using a syringe pump (RWD Life Science Co, China) at the flow rate that generated a wall shear stress of 2 dynes/cm^2^, which represents the shear stress (0.5–4 dynes/cm^2^) in venous circulation [32]. Cell adhesion were monitored at 20 min after perfusion using the inverted fluorescence microscope(NiKon Diaphot 330, Japan). Afterwards, PBS was perfused at 2dynes/cm^2^ for 10 min to remove non adherent cells.Strongly adhered cells in more than five different fields were recorded under a phase-contrast inverted fluorescence microscope (NiKon Diaphot 330, Japan). For the antibody blocking experiments, three breast cancer cells (MDA-MB-231 cells, HS-578 T cells and MCF-7 cells) were treated with a rabbit anti-human TF polyclonal antibody (20 μg/mL), a rabbit anti-human fibronectin polyclonal antibody (10 μg/mL) or HUVECswith a rabbit anti-human β_1_integrin polyclonal antibody (1:100 dilution), a mouse anti-human α_3_ integrin polyclonal antibody(10 μg/mL), a rabbit anti-humanE-selectin polyclonal antibody(10 μg/mL) for 30 min prior to the assay at 37°C. The pretreated cells were rinsed two times in culture media before being perfused over the HUVECs through the flow chamber. For the TF stimulation assay, recombinant human soluble TF (rTF), which lacks the cytoplasmic and transmembrane domain (PeproTech,150–19) depends on the plasma concentration of the patients with breast cancer [33]. HS-578 T cells belong to TNBC and express low levels of TF, herein, we chosen HS-578 T cells were treated with recombinant TF for 24 hrs at 37°C. For rTF in vitro antagonism experiments, HUVECs were treated with rTF (100 ng/mL) for 4h at 37°C, cell adhesion were prepared according to the static adhesion assay procedure indicated above.

### HUVECs adhesion assay

The HUVECs adhesion assay was referenced previous study [28], 96-well tissue culture plates were coated by rTF (100 ng/well), 10% BSA (100 μL/well) served as control. HUVECs were treated by PBS or β_1_integrin antibody (1:100 dilution, GeneTex, GTX128839) 30 min at 37°C, 2 × 10^4^ HUVECs/well were added to 96-well plates under 5% CO2 at 37 °C for 2hrs, PBS washed the non-adhesion cells two times, three random fields exclude the edges were counted by under a phase-contrast inverted microscope (NiKon Diaphot 330, Japan), and then 10 μL CCK8 (YEASEN, 40203ES60) was added to the plates incubated at 37°Cfor 2hrs. The optical density (OD) values were read at 460 nm using a Bio-Rad 680 microplate reader (Bio-Rad Laboratories, Hercules, CA).

### Cell viability assay

Cells (8 × 10^3^/well) were seeded in a 96-well plate and cultured overnight at 37°C. They were then cultured for 24hrs in the medium that contained individual testing agents at 37°C in the absence and presence of either TF antibody (20 μg/mL), fibronectinantibody (10 μg/mL), β_1_integrin antibody (1:100), α_3_integrin (10 μg/mL), E-selectin (10 μg/mL). Cell viability was measured by the CCK8 assay. Briefly, 10 μL CCK8 was added to the 96-well plates incubated at 37°C for 2hrs. The optical density (OD) values were read at 460 nm using a Bio-Rad 680 microplate reader (Bio-Rad Laboratories, Hercules, CA).

### Statistical analysis

Data were analyzed using GraphPad prism 5.0. Quantitative results were recorded as mean ± SD. Statistical comparisons of the mean were performed using t-test or the analysis of variance (ANOVA), as discussed for individual datasets. A *P* value of < 0.05 was considered statistically significant.

## Results

### TF expression on breast cancer cells

**Figure 1.**
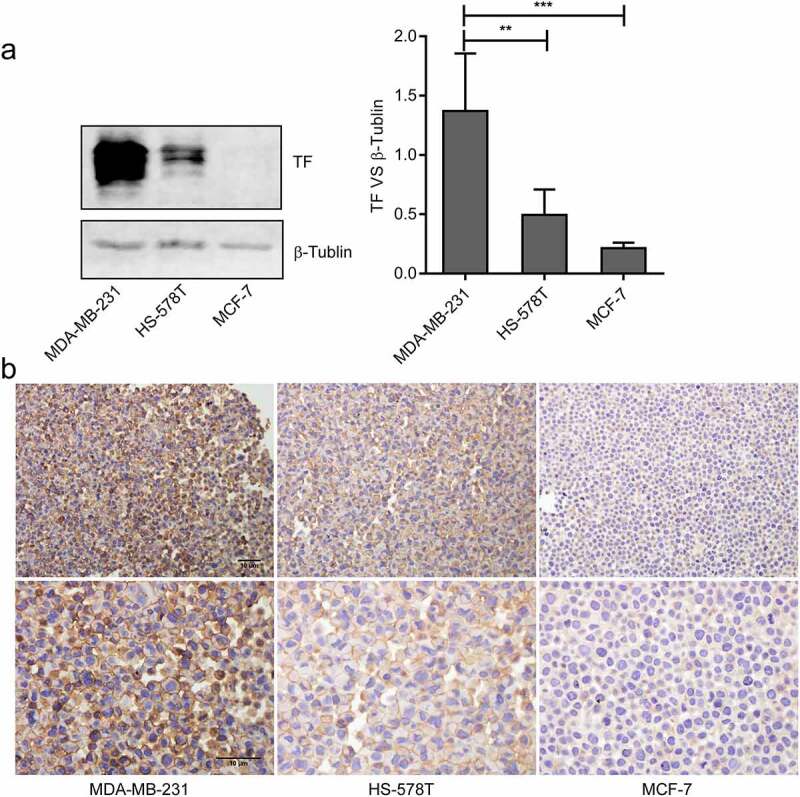
**The expression and location of TF in breast cancer cells**.(a)The levels of TF in MDA-MB-231cells, HS-578 T cells and MCF-7 cells were monitoredusing Immunoblots(n = 3),the bar graphon the right represents densitometry measurements for multiple SDS-PAGE(**P < 0.01, ***P < 0.001 vs.MDA-MB-231 cells). (b) Cell immunohistochemistry was used to measure the expression of TF in MDA-MB-231 cells, HS-578 T cellsand MCF-7 cells, bar = 10 μm

A high level of TF expression was detected on MDA-MB-231 cells by immunoblots compared with HS-578 T cells and MCF-7 cells (Figure 1a). Furthermore, TF was detected on the surface and cytoplasm of MDA-MB-231 cells and HS-578 T cells (Figure 1b).

### Effect of TF on MDA-MB-231 adhesion to HUVECs

**Figure 2.**
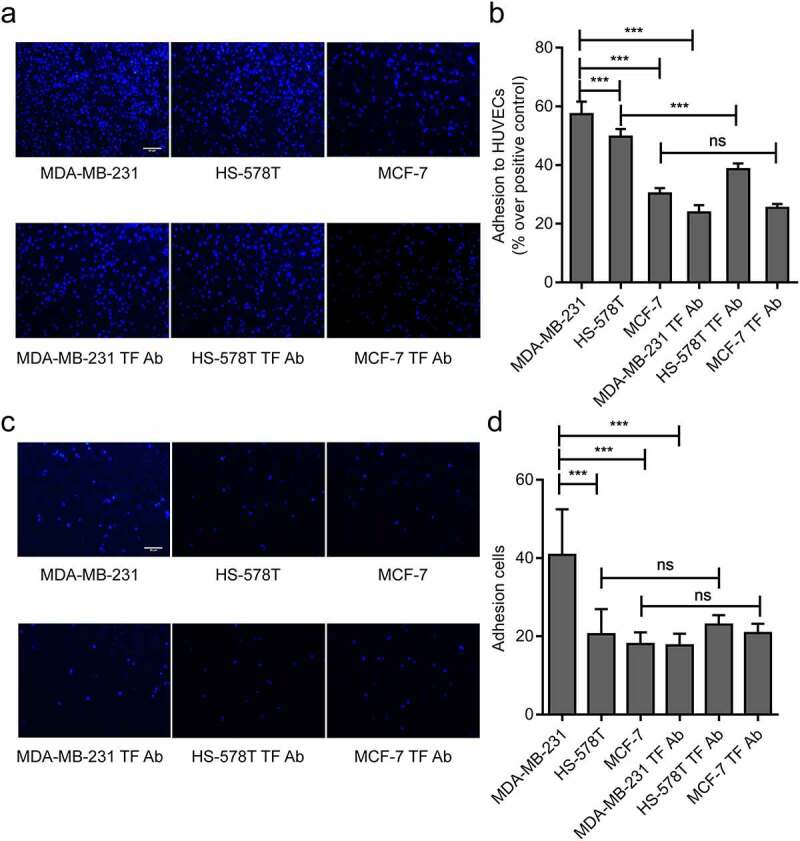
**Effect of endogenous TF on MDA-MB-231 adhesion to HUVECs**. (a, b)Three breast cancer cells (MDA-MB-231 cells, HS-578 T cells and MCF-7 cells) adhere to HUVECs wereexamined by static adhesion assay, and the adhesion of three breast cancer cells to endothelial cells blocked by TF antibodywere examined by static adhesion assay, bar = 20 μm, the bar graph represents the adhesion rate ((n = 4, ***P < 0.001 vs. MDA-MB-231 cells, no significant (ns). (c, d) Three breast cancer cells (5 × 10^5^ cells/mL) adhere to endothelium under shear (2dynes/cm^2^)were examined by Parallel plate flow assay, and the adhesion of three breast cancer cells to endothelial cells blocked by TF antibody were examined usingParallel plate flow assay, bar = 20 μm, the number of adhesion cells was quantified by Image J (right, n ≥ 5, ***P < 0.001 vs. MDA-MB-231 cells, (ns (no significantly)

Two methods were used to measure the levels of TF expression and their impact on the adhesion of these cells to HUVECs. The number of adherent cells was significantly higher for MDA-MB-231 cells than HS-578 T and MCF-7 cells to cultured HUVECs under both static and flow conditions, the enhanced adhesion of MDA-MB-231 cells to HUVECs was blocked significantly by a TF antibody under both static and flow conditions (representative videos are shown on Suppl. videos 1 and 2). The adhesion of HS-578 T cells to HUVECs was partially blocked by a TF antibody under static conditions, but not under flow conditions. In contrast, the TF antibody had a minimal impact on the adhesion of MCF-7 cells to HUVECs under both static conditions(Figure 2(a, b) and flow conditions (Figure (2c, d). These data suggest that TF mediated the adhesion of MDA-MB-231 cells to HUVECs.

### Exogenous TF enhanced adhesion of HS-578 T to HUVECs

**Figure 3.**
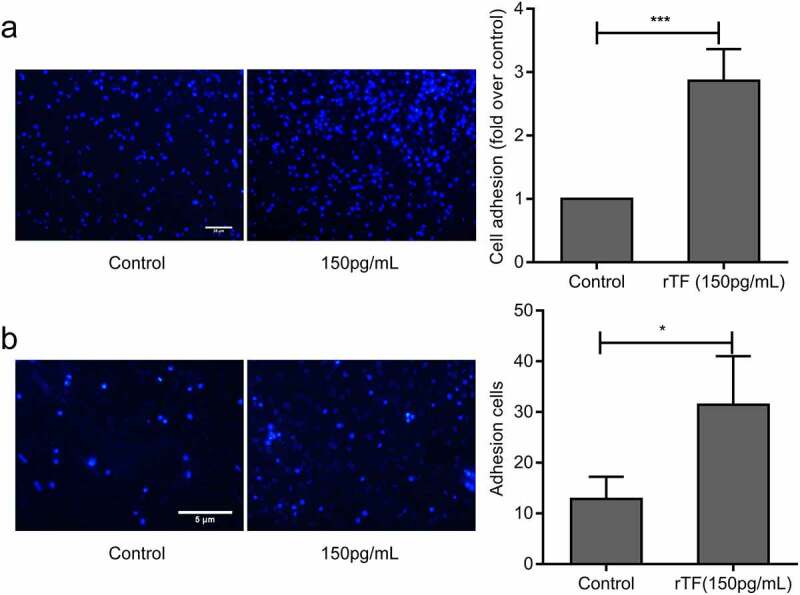
**Effect of Exogenous TF on adhesion of HS-578 T to HUVECs**.(a) HS-578 T pretreated with 150pg/mLrTF, the adhesion of cells to HUVECs were examined by static adhesion assay,bar = 20 μm, the bar graph on the right represents the adhesion rate (n = 6, **P < 0.01 vs. untreated cells).(b) The adhesion of HS-578 T cells(5 × 10^5^cells/mL) pretreated with 150 pg/mL rTF to endothelium under shear (2dyns/cm^2^)were examined by Parallel plate flow assay, bar = 5 μm, the number of adhesion cells was quantified by Image J (right,n ≥ 5, *P < 0.05 vs. untreated cells)

The results presented in Figure 2 suggest that endogenous TF mediated the adhesion of MDA-MB-231 cells to HUVECs. When HS-578 T cells, which expressed a low level of TF, were pretreated with recombinant human soluble TF for 24 hrs, their adhesion to HUVECs was significantly increased (Figure 3a). The exogenous TF also promoted the adhesion of HS-578 T cells to HUVECs under flow conditions(Figure 3b). These data suggested that exogenous TF enhanced adhesion of HS-578 T cells to HUVECs.

### TF depended on β_1_ integrin to mediate the adhesion of MDA-MB-231 cells to HUVECs

**Figure 4.**
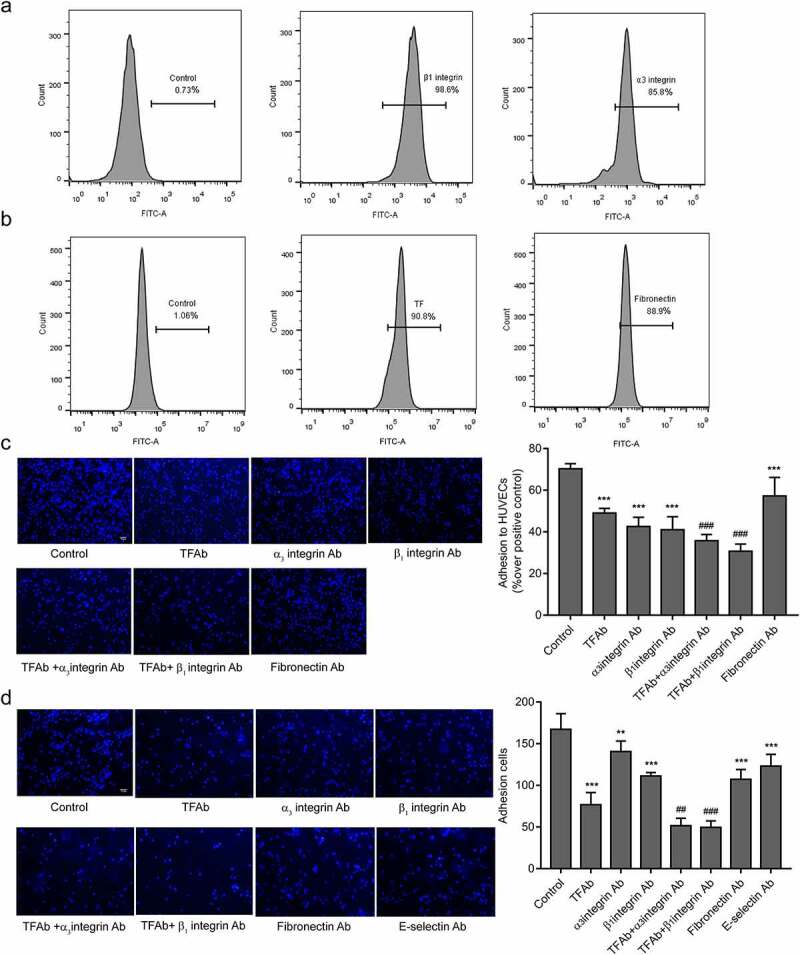
**Effect of TF and** α_3_β_1_ **integrin on the MDA-MB-231cells adhesion to HUVECs.**

**Figure 5.**
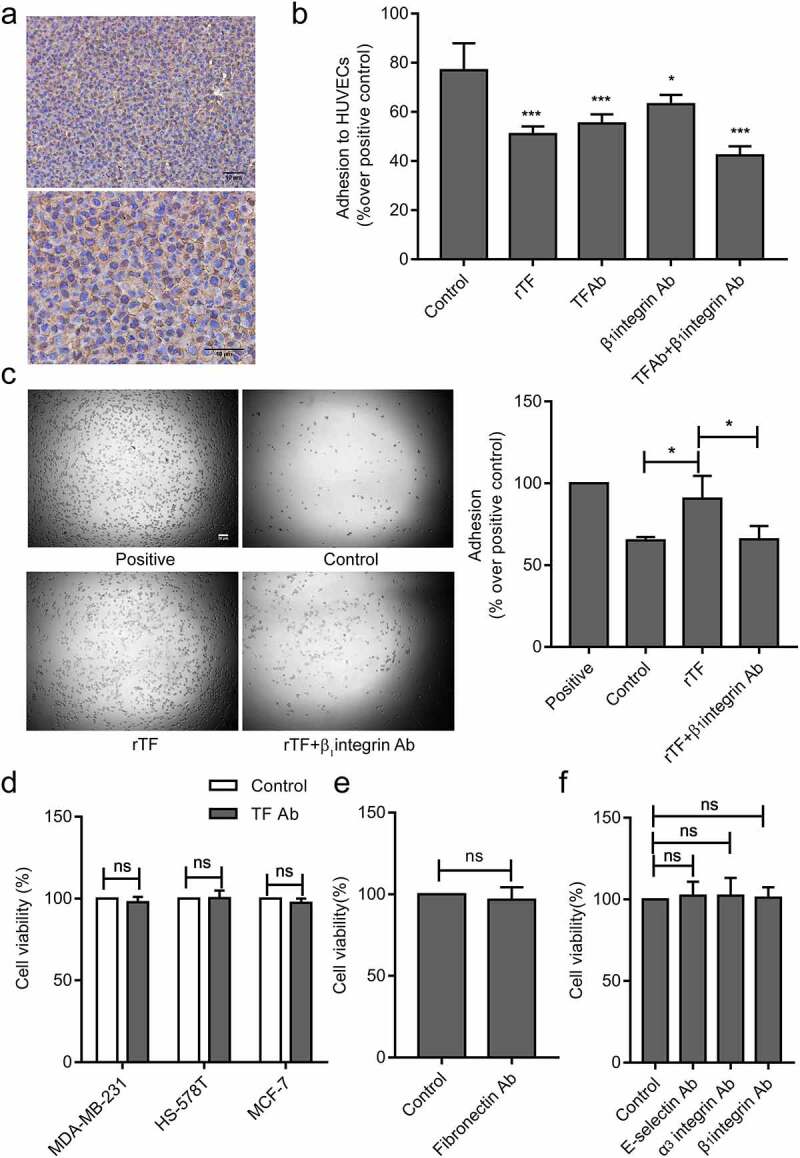
rTF depended onβ_1_ integrin on HUVECs

The endothelial integrin α_3_β_1_ mediates the adhesion of tumor cells to endothelial cells [29]. In order to explore the molecular mechanism of TF-mediated tumor cells adhesion to HUVECs, we hypothesized that TF expressed on tumor cellsrelies on β_1_integrin expressed on HUVECs.β_1_andα_3_ integrin were detected on the surface of HUVECs (Figure 4a, 5a).The adhesion of MDA-MB-231cells to HUVECs were significantly blocked by β_1_or α_3_integrin antibody and synergistically by a combination of TF antibody and β_1_or α_3_integrin antibody under static (Figure 4c) and flowconditions (Figure 4d) .α_3_β_1_ integrin are receptors for fibronectin [34].The high levels of TF and fibronectin were detected on the surface of MDA-MB-231cells (Figure 4b), and cell adhesion was partial blocked by fibronectin antibody (Figure 4c,d). As it has been described that E-selectin mediates the adhesion of tumor cells on endothelial cells [35]. Blocking of E-selectin alone resulted in reducing MDA-MB-231cells adhesion to HUVECs under flow conditions(Figure 4d). Cell-cell adhesion was partial blocked when HUVECs were pretreated by rTF under static conditions (Figure 5b).We observed an increased number of adhesion cells after HUVECs added into rTF coating plates by HUVECs adhesion assay 2 h, and the adhesion of HUVECs pretreated with β_1_integrin antibody significantly decreased (Figure 5c),and we examined the effect of antibodies against TF, fibronectin, E-selsectin, α_3_ and β_1_integrin, no antibody above affected the viability of MDA-MB-231 cells, HS-578 T cells, MCF-7 cells and HUVECs in the experimental setting (Figure 5d-f).

(a)Expression of adhesion molecules (β_1_integrin, α_3_ integrin) on HUVECs were analyzed by flow cytometry. (b) Expression of TF and fibronectin on MDA-MB-231cells were analyzed by flow cytometry. (c)The effect of TF and α_3_β_1_integrin on the cell-cell adhesion between MDA-MB-231cells and HUVECswas examined by static adhesion, MDA-MB-231 cells(4 × 10^5^cells/mL) pretreated with TF antibody (20 μg/mL) and fibronectin antibody(10 μg/mL) respectively, HUVECs pretreated with β_1_integrinantibody (1:100 dilution) and α_3_ integrin antibody(10 μg/mL) respectively,bar = 10 μm. The bar graph right represents the adhesion rate (n = 5, ***P < 0.001 vs. untreated cells, ### *P* < 0.001 vs.TF Ab).(d) The effect of TF and α_3_β_1_integrin on the cell-to-cell adhesion between MDA-MB-231cellsand HUVECswas examined by Parallel plate flow assay, MDA-MB-231 cells(1 × 10^6^cells/mL) pretreated with TF antibody (20 μg/mL) and fibronectin antibody(10 μg/mL) respectively, HUVECs pretreated with β_1_integrinantibody (1:100 dilution), α_3_ integrin antibody(10 μg/mL) and E-selectin (10 μg/mL) respectively,bar = 10 μm. The bar graph right represents the number of adhesion cells was quantified by Image J(n ≥ 5, **P < 0.05, ***P < 0.001 vs. untreated cells, ### *P* < 0.001 vs.TF Ab).

(a) Cell immunohistochemistry assay analysis of the expression and location of β_1_integrin in HUVECs, bar = 10 μm. (b) The effect of rTF treated HUVECs on cell-cell adhesion under static condition, HUVECs were treated by rTF(100 ng/mL) for 4 h, the adhesion between MDA-MB-231cells and HUVECs was examined by static adhesion assay,(n = 4, *P < 0.05,***P < 0.001 vs.untreated groups).(c)HUVECs adhesion to rTFcoating surface was examined by HUVECs adhesion assay, bar = 20 μm, the bar graph right represented adhesion rate was examined by CCK8(n = 3, *P < 0.05 vs.rTF group. Positive group: no washing; Control group: no rTF protein coating; rTF group: rTF protein coating; rTF+β_1_integrin antibody group: HUVECs treated by β_1_integrin antibody adhesion to rTF protein coating). (d) CCK8 assay analysis the viability of breast cancer cells (MDA-MB-231, HS-578 T and MCF-7cells) pretreated with TF antibody (n = 5, ns vs. control cells). (e)CCK8 assay analysis the viability of MDA-MB-231cells pretreated with fibronectinantibody, (n = 5, ns vs. control cells).(f) CCK8 assay analysis the viability of HUVECs pretreated with α_3_ integrinantibody, β_1_integrinantibody and E-selectin antibody (n = 5, ns vs. control cells).

## Discussion

The interaction between cancer cells and the vascular endothelium is a widely recognized step for cancer metastasis, but the underlying mechanism for the interaction has not been fully [36]. This study was designed to investigate the role of cancer cell-derived TF in mediating the adhesion of breast cancer cells to HUVECs. Our results showed that TF expressed onMDA-MB-231 cells andEC-derived β_1_integrin promote synergistically the cancer cell-EC interaction.

Cell adhesion to endothelial cells has been traditionally studied under static conditions where these cells were incubated together for a prolonged period of time that is unlikely to occur in rapidly flowing blood. We therefore examined the ability MDA-MB-231 cells to adhere to HUVECs under flow that generated venous shear stress. This experiment allowed us to distinguish transient and permanent adhesion of cells in real time in the condition mimicking the vasculature [37,38]. We observed that MDA-MB-231 cells expressing high TF levels adhered strongly to HUVECs under static and venous flow condition. A TF antibody partially blocked the adhesion of MDA-MB-231 cells to endothelial cells. The findings suggest that TF and other molecules on the surface of these cells are involved in the cell-cell interaction. This is supported by the finding that exogenous soluble TF promoted the adhesion of HS-578 T cells, which expressed low levels TF, to endothelial cells. However, the molecule(s) that the soluble TF bound to cancer cells remains to be identified.

Leukocyte cell adhered to vascular endothelium including leukocyte rolling, arrest, firm adhesion and migration requires several cell adhesion molecules (CAMs), which including selectins, integrins, and immunoglobulin superfamily [39]. Selectins mediate initial cell adhesion like leukocyte rolling [40], such as L-selectin expressed on leukocytes, E-selectin expressed on endothelial cells and P-selectin expressed on endothelial cells and platelets [41]. We analyzed the adherence properties of MDA-MB-231cells on E-selectin blocked HUVECs. The data showed that blocking against E-selectin on HUVECs decreased the adhesion between MDA-MB-231 cells and HUVECs. We speculate that E-selectin also may induce a stronger adherence to the endothelial cells [12]. Whether TF is involved in mediating the rolling and adhesion of tumor cells and endothelial cells through E-selectin requires further research.

In this study, it was focused that the role of TF in the process of firm adhesion. It has been shown that endothelial integrins mediate cancer cells adhesion to endothelial cells. Instead of mediating transient interaction mediated by selection and carbohydrates, integrins promote the firm adhesionof cancer cells to endothelial cells, the process necessary to the transendothelial migration of cancer cells to the extracellular space [42]. α_3_β_1_ integrin has been regarded as the receptor of fibronectin [34]. After blocking fibronectin in MDA-MB-231 cells, cell-cell adhesion decreased partially. Further show that α_3_β_1_ integrin take part in the interaction between tumor cells and endothelial cells. Previous studies have reported that cross-talk of TF with α_3_β_1_ integrins regulates cell migration [43]. We found that α_3_ and β_1_ integrin on endothelial cell surface participates in the adhesion of tumor cells to endothelial cells mediated by TF. Our results are consistent with reports.

We have indeed found that MDA-MB-231 cells firmly adhered to ECs in a TF and β_1_integrin-dependent manner. The question remains as whether TF-β_1_integrin interaction triggers intracellular signaling event in cancer cells, endothelial cells or both to alter the ability of these cells to migrate, as the ability of TF to promote cancer cell migration and transendothelial metastasis [43,44]. It has been previously reported that exogenous soluble tissue factor or tissue factor on extracellular vesicles induces intracellular signaling to promote the proliferation of endothelial cells and this effect is mediated through β_1_integrin [45], this TF-β_1_integrin induced signaling could potentially promote tumor angiogenesis.

In conclusion, our study indicates that TF on breast cancer cells surface may facilitate their adherence to HUVECs bycooperating with β_1_integrin on HUVECs, thus, inhibition of tissue factor mediated tumor cells adhesion to endothelium could represent a potential new target of cancer metastasis.

## Supplementary Material

Supplemental Material
